# Extracellular vesicles: emerging roles, biomarkers and therapeutic strategies in fibrotic diseases

**DOI:** 10.1186/s12951-023-01921-3

**Published:** 2023-05-24

**Authors:** Junyan Zhu, Sicong Wang, Dakai Yang, Wenrong Xu, Hui Qian

**Affiliations:** Jiangsu Province Key Laboratory of Medical Science and Laboratory Medicine, Department of Laboratory Medicine, School of Medicine, Jiangsu University, Zhenjiang, Jiangsu 212013 China

**Keywords:** Extracellular vesicles, Fibrosis, Pathogenesis, Diagnosis, Therapy

## Abstract

Extracellular vesicles (EVs), a cluster of cell-secreted lipid bilayer nanoscale particles, universally exist in body fluids, as well as cell and tissue culture supernatants. Over the past years, increasing attention have been paid to the important role of EVs as effective intercellular communicators in fibrotic diseases. Notably, EV cargos, including proteins, lipids, nucleic acids, and metabolites, are reported to be disease-specific and can even contribute to fibrosis pathology. Thus, EVs are considered as effective biomarkers for disease diagnosis and prognosis. Emerging evidence shows that EVs derived from stem/progenitor cells have great prospects for cell-free therapy in various preclinical models of fibrotic diseases and engineered EVs can improve the targeting and effectiveness of their treatment. In this review, we will focus on the biological functions and mechanisms of EVs in the fibrotic diseases, as well as their potential as novel biomarkers and therapeutic strategies.

## Introduction

Extracellular vesicles are natural membrane-enclosed particles and encompass many subtypes according to their origin and biogenesis [1]. The vesicles directly budding from the plasma membrane (PM) are termed microvesicles (MVs, 100-1000 nm)[2]. Small vesicles forming in multivesicular endosomes or internal multivesicular bodies (MVB) before released by exocytosis are designated as “exosome” (30-150 nm)[3]. Another major kind of EVs is apoptotic bodies, a subset of lager subcellular particle (1–5 μm) released after programmed cell death [4]. MVs, exosomes and apoptotic bodies are generally considered three traditional classes of membranous vesicles, while many distinct subgroup components and cell derivatives are gaining increasing attention, such as exomeres [5, 6] and supermeres [7]. Various names appeared in different research according to different sizes, cargo contents and biogenesis, including shedding vesicles, microparticles, ectosomes and apoptotic blebs [8, 9]. There were no standard EV nomenclature for a long time until International Society for Extracellular Vesicles (ISEV) recommended using the umbrella term “extracellular vesicles” to refer to all types of membrane-derived vesicles and classifying them into small EVs (sEVs) and medium/large EVs (m/lEVs) based on their size (MISEV2018)[10]. Thus, EVs are the predominant term in this review unless specific designations are used in some study.

**Fig. 1:**
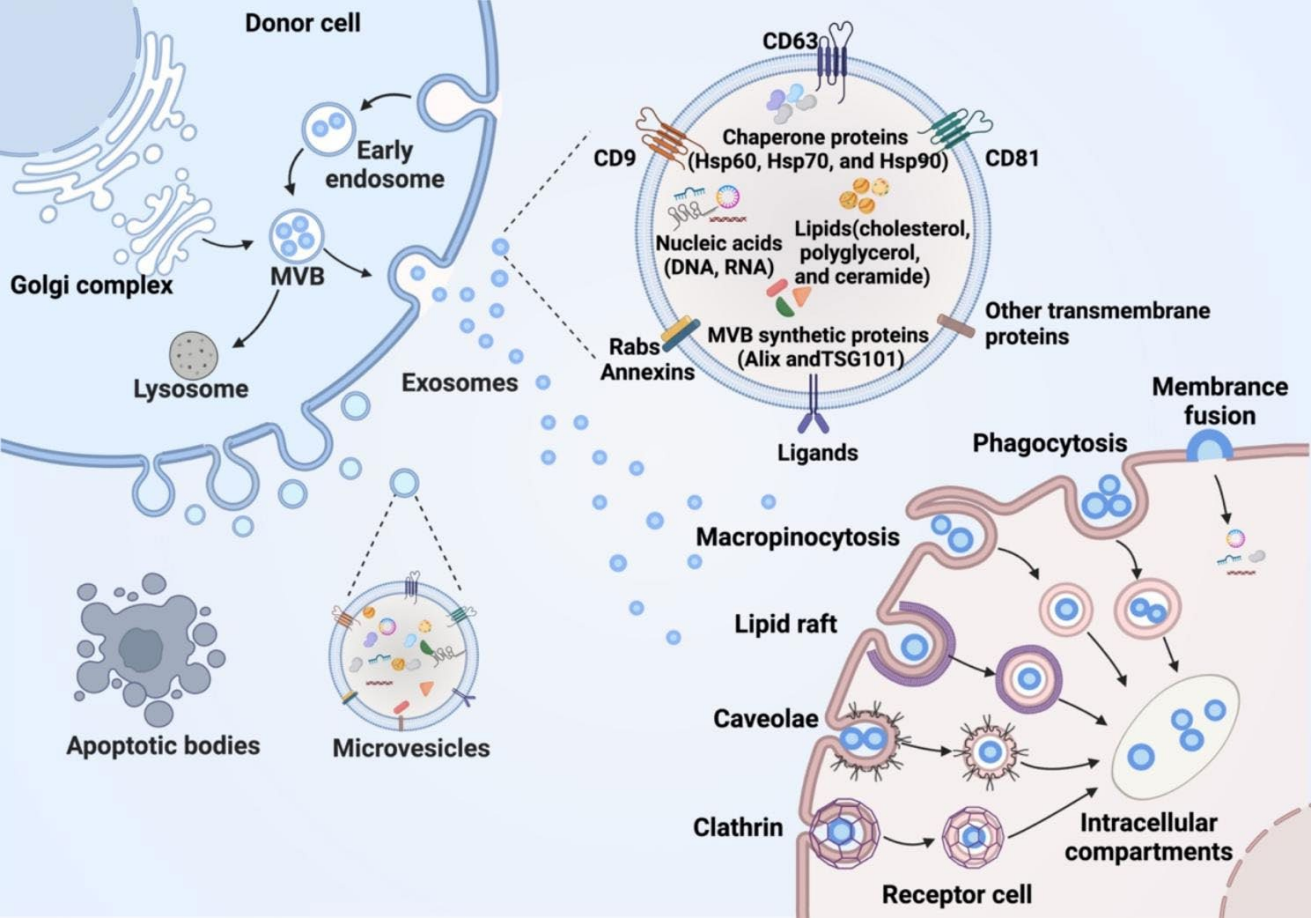
EV classification, biogenesis, and uptake. Apoptotic bodies and microvesicles are generated by directly budding from the plasma membrane, while exosomes are formed by endosomal pathway. Coasted by phospholipid bilayer, EVs are good carriers for bioactive cargos, including proteins, lipids, and nucleic acids. EVs can be internalized by receptor cells through membrane fusion, phagocytosis, micropinocytosis, clathrin-dependent endocytosis, caveolin-mediated endocytosis, or lipid raft-mediated endocytosis

Almost all types of cells can release EVs and they harbor common features such as certain transmembrane proteins (CD9, CD63, and CD81), chaperone proteins (Hsp60, Hsp70, and Hsp90), MVB synthetic proteins (Alix and TSG101), membrane transport/fusion proteins (Annexins and Rabs), lipids (cholesterol, polyglycerol and ceramide), and nucleic acids (DNA, mRNA, microRNA, lncRNA and circular RNA)[11, 12]. Besides non-specific composition, EVs contain many parent-dependent contents reflecting the physiological state of origin cell and tissue. Once released by donor cells, EVs can be selectively taken up by target cells via membrane fusion or multiple endocytic pathways, including phagocytosis, micropinocytosis, clathrin-dependent endocytosis, caveolin-mediated endocytosis and lipid raft-mediated endocytosis [13]. Recipient cells respond to EV-loaded cargos when EVs are internalized, allowing parent cell signals to be transmitted to adjacent or distant cells without direct cellular contact [14] (Fig. 1). Accumulating evidence suggests that EVs show great potential for novel biomarkers of diseases, therapeutic agents and ideal carriers for drug delivery due to their unique biological characteristics [15, 16].

In recent years, studies have confirmed that EV plays an important role in many diseases, especially in fibrotic diseases. Fibrosis is usually associated with tissue damage, during which period a variety of cells, such as epithelial cells, endothelial cells, immune cells, and fibroblasts, contribute to wound healing through complicated cellular interaction [17]. After initial injury, hemostasis takes place and thrombin contribute to the formation of early fibrin network. The ensuing inflammation activates fibroblasts into myofibroblasts, which produce a large amount of extracellular matrix (ECM, collagen and fibronectin) and contraction force, inducing the wound remodeling [18, 19]. In certain instances, this process may degenerate into fibrosis as a result of persistent activation of myofibroblasts, which promote excessive production and accumulation of ECM proteins. Fibrosis can be observed in multiple organs, including the lung, liver, kidney, and heart [20], ultimately leading to severe destructive structural remodeling of late diseased tissues or organs [21]. However, the precise mechanism of fibrosis remains uncertain, and hinders the development of targeted therapy. Here, we elaborate on the EV biological functions, and their potential as biomarkers for fibrosis. We also discuss the recent advance in the application of EVs in the treatment of fibrotic diseases.

## Mechanisms of EVs in fibrotic diseases

EVs are now demonstrated to mediate physiological and pathological processes of cells, organs, and organisms throughout the body. The cargos of EVs may reflect the state of the parent cells, body metabolic and pathologic condition, having the potential value for clinical diagnostics [22] (Table 1). Aberrant cell maps and dysregulated cell-to-cell communication have been uncovered in many fibrotic organs by single-cell sequencing and considered as the important causes of fibrosis [23–25], during which process EV-mediated intercellular signal transduction are various due to differences of microenvironment and structure between these organs. Identifying the unique role of EVs in fibrotic diseases will be critical to utilizing them effectively in the future.

**Table 1:** EVs as biomarkers for fibrotic diseases

Fibrotic organ	Disease	Source of EVs	EV Cargos/Markers	Expression	Ref.
Liver	biliary cholangitis /biliary atresia	serum	lncRNA H19	↑	[38, 39, 41]
	HCV	plasma	miR204-5p, miR181a-5p, miR143-3p, miR93-5p and miR122-5p/DIAPH1	↓/↑	[42]
	NAFLD	plasma	CD14^+^/CD16^+^	↓	[43]
	liver cirrhosis	plasma	CD31/41^−^	↑	[44]
	liver cirrhosis	plasma	AV^+^	↓	[45]
Lung	IPF	BALF	miR-142-3p, miR-33a-5p/ let-7d-5p	↑/↓	[59]
	IPF	BALF	CD56, CD105, CD142, CD31 and CD49e	↑	[60]
	IPF	BALF	WNT-5a	↑	[50]
	IPF	urine	miR-let-7d, miR-29a-5p, and miR-181b-3p/ miR-199a-3p	↓/↑	[62]
	MVPF	BALF	ncRNAs	undefined	[61]
Heart/Vessel	HF	plasma	miR-425, miR-744	↑	[75]
	atherosclerosis	plasma	miR-23a-3p, miR-92a-3p	↑	[71, 76]
	AF	serum	lncRNA MIAT	↑	[77]
	senescence	serum	HSP70	↓	[78]
Kidney	CKD	urine	miRNAs and mtRNAs	undefined	[102, 103]
	CKD	urine	circ_0036649	↓	[104]
	glomerular disease	urine	circ_0008925	↑	[105]

### Liver fibrosis

#### Pathological effects of EVs in liver fibrosis

Liver fibrosis occurs in most types of chronic liver diseases, including chronic hepatitis B virus (HBV) or hepatitis C virus (HCV) infection, alcoholic liver disease, non-alcoholic fatty liver disease (NAFLD) and steatohepatitis (NASH). Advanced liver fibrosis results in hepatic cirrhosis, liver failure, and often requires liver transplantation in the end-stage [26].

Hepatocytes are damaged at the initial of liver disease as targets of most hepatotoxic agents. For example, in the course of NAFLD, EVs secreted by endoplasmic reticulum stress-induced adipocytes promote glycerol and triglycerides accumulation in hepatocytes through the delivery of Aldo-keto-reductase 1b7 (Akr1b7) thus trigger NASH [27]. Damaged and dead hepatocytes release pathogenetic mediators, leading to inflammatory cells recruitment and hepatic stellate cells (HSCs) activation. Hirsova et al. [28] reported that in NASH lipotoxic hepatocytes activate macrophage and mediate inflammation by secreting tumor necrosis factor-related apoptosis-inducing ligand (TRAIL) bearing EVs. A paper by Seo et al. [29] demonstrated an indirect immunomodulatory effect of hepatocyte derived EVs in a carbon tetrachloride (CCl4) treated mouse model, where they activate toll-like receptor 3 (TLR3) in HSCs and thus enhance interleukin-17 A (IL-17 A) produced by γδ T cells, exacerbating liver fibrosis. However, most studies focus on describing how EVs from hepatocytes modulate HSCs behavior and promote liver fibrosis. In an alcoholic hepatitis mouse model, hepatocytes release increased numbers of EVs, which can activate HSCs via a set of miRNAs [30]. Another study showed that mannan-binding lectin serine protease 1(MASP1) is enriched in hepatocyte derived EVs and activates HSCs through the p38 MAPK/ATF2 signaling pathway to promote liver fibrogenesis [31]. In addition, overloaded iron can be shuttled from hepatocytes to HSCs via EVs, and the aberrant iron distribution stimulates HSCs activation in a NAFLD/NASH model [32]. Although the mechanisms by which hepatocyte derived EVs modulate HSCs and the cargo delivered by these EVs are diverse across different studies, they all demonstrated the importance of EV-mediated hepatocyte-HSC crosstalk in fibrotic liver.

Activated HSCs produce large amounts of ECM proteins and insoluble collagen, which is the primary cause of liver fibrosis [33]. Besides hepatocytes, immune cells also participate in the activation of HSCs during hepatic fibrosis. For instance, macrophage stimulated by lipopolysaccharide deliver miR-103-3p via EVs and promote HSCs proliferation and activation [34]. Moreover, activated HSCs can release EVs enriched with fibrogenic proteins by decreasing autophagic activity [35]. Some pathogenic cargos enriched in EVs derived from activated HSCs have been revealed, such as PDGFRα[36], GLUT1 and PKM2 [37], which may be new targets for the treatment of liver fibrosis.

In the context of cholestasis-induced liver fibrosis, enriched non-coding RNA-H19 (LncRNA-H19) carrying EVs released from cholangiocytes can be internalized by both hepatocytes and HSCs, where they promote liver injury by down regulating small heterodimer partner (SHP) and increasing ECM formation [38, 39]. Additionally, these H19-carrying EVs enhance the M1 polarization of Kupffer cells, further exacerbating hepatic inflammation [40]. These findings underscore the multiple roles of cholangiocytes in the pathogenesis of liver fibrosis.

**Fig. 2:**
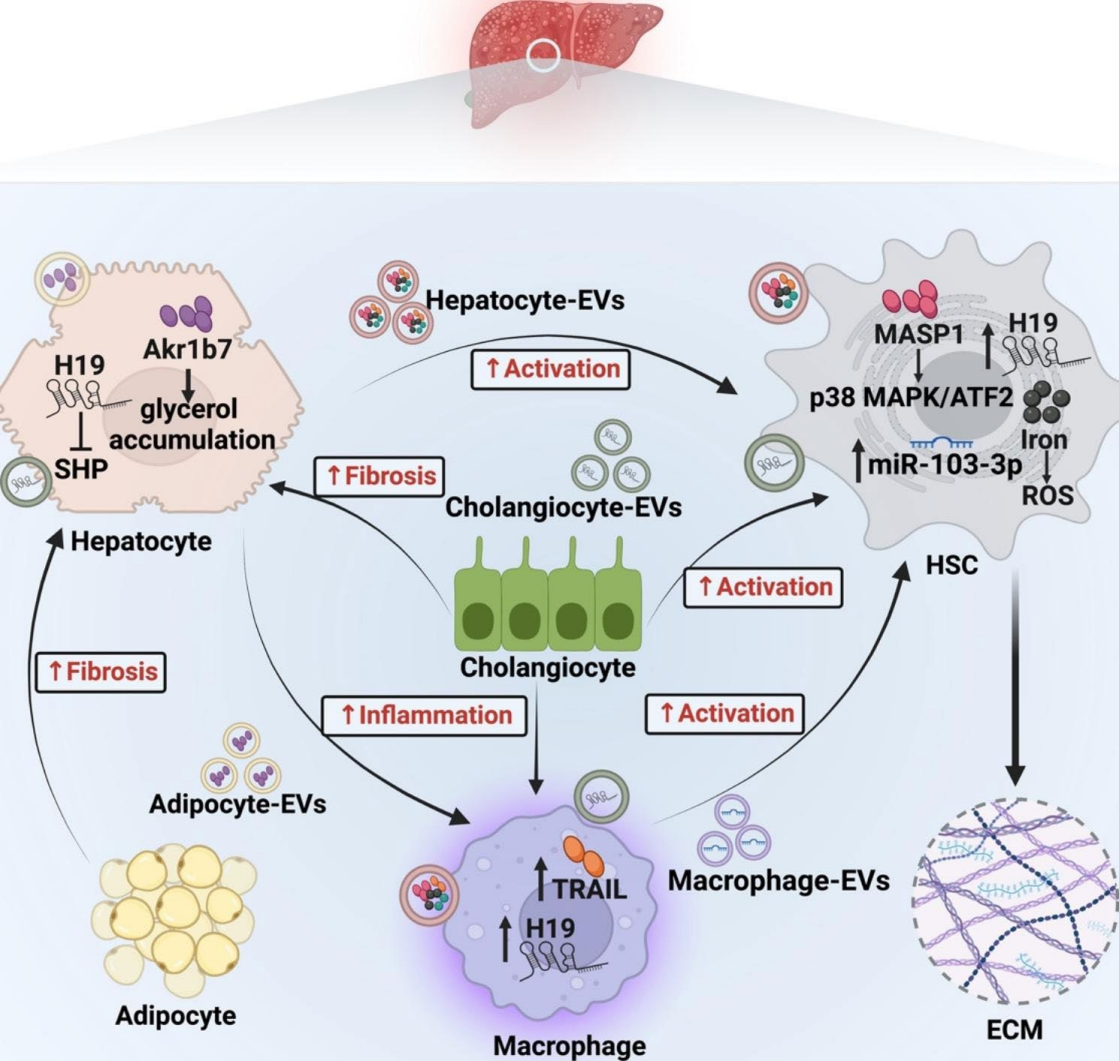
EV-mediated intercellular communication in liver fibrosis. In fibrotic liver, hepatocytes, HSCs, macrophages, cholangiocytes, and adipocytes communicate through vesicles carrying bioactive molecules and contribute to the disease progression

Overall, EVs from hepatocytes, immune cells, HSCs, and cholangiocytes shuttle across intercellular space and transfer “pathogenic molecules” among different liver cells, contributing to progression of fibrosis in liver (Fig. 2). HSCs, as the primary source of ECM protein, are the hub of cellular communication networks in fibrotic liver. Thus, reducing EV-based signal transduction toward HSCs and inhibiting their activation may be the key point for liver fibrosis therapy.

#### EVs as diagnostic and prognostic biomarkers in liver fibrosis

The value of EVs as biomarkers for liver fibrosis are underlying widely exploration. In biliary cholangitis and biliary atresia patients, the level of EV-carried H19 in serum has been reported to be correlated to the severity of fibrotic liver injury [38, 39, 41]. EVs in plasma of patients infected with HCV have been purified and studied systematically. Montaldo et al. [42] found that EVs derived from HCV-infected patients before and after direct-acting antiviral therapy contain lower levels of antifibrogenic miRNAs (miR204-5p, miR181a-5p, miR143-3p, miR93-5p and miR122-5p) but more profibrogenic proteins (DIAPH1) compared with healthy donors. This finding also indicates that the constant changes in EV composition might be responsible for long-term fibrosis progress. In addition to cargo contents, changes in the amount of EVs with different surface markers are related to the progression of liver fibrosis. A preliminary cohort study has revealed that CD14 and CD16 positive (CD14+/CD16+) leukocyte EVs counts in plasma are inversely associated with liver fibrosis severity and show potential to improve risk prediction of severe fibrosis in NAFLD [43]. Further, Payancé et al. [44] have reported that circulating hepatocyte EVs levels effectively improve prediction of 6-month mortality in patients with advanced chronic liver disease. Moreover, decreased plasma level of annexin V-positive (AV+) platelet-derived EVs has been proved to be correlated with significantly lower transplant-free survival in cirrhotic patients [45].

### Lung fibrosis

#### Pathological effects of EVs in lung fibrosis

Pulmonary fibrosis (PF) can be triggered by a variety of causes, such as allergens, irritant chemicals, radiation, pathogenic microorganisms, and environmental particles [46]. Featured by epithelial cells injury, fibroblast activation and ECM deposition, idiopathic pulmonary fibrosis (IPF) is one of the most common pulmonary fibrotic diseases, but its aetiology remains unknown [47]. Recent research has discovered that senescent bronchial epithelial cells (BECs) transfer senescence to neighboring healthy cells via miRNA loaded EVs, resulting in a feed-forward cycle of senescence within epithelium and aggravating IPF state [48]. In a mouse model with PF, pulmonary vascular endothelial cells released EVs have been reported to contain low levels of microRNA let-7d and trigger lung pericyte fibrosis though TGF-β1/Smad and Wnt/β-catenin signaling cascades [49]. Although not predominant ECM-producing cells, these lung resident cells displaying fibrogenic changes occupy a vital position in progression of lung fibrosis by creating a pathogenic microenvironment.

Numerous studies focus on the EV-mediated fibroblasts activation in fibrotic lung. Increased EVs have been observed in bronchoalveolar lavage fluid (BALF) from patients with IPF, which carry signaling mediators such as WNT-5 A and drive fibroblast proliferation [50]. Similarly, Zhu et al. [51] claimed that in PF rat model there is abundant miR-204-5p in BALF derived EVs, which can potentiate PF progression and activate fibroblast experimentally by targeting AP1S2. However, the specific source of these profibrotic EVs in BALF has not been demonstrated clearly. Combined with both positive and negative regulatory effects, macrophages are considered as important mediator in PF [52, 53]. Overexpressed miR-328 and miR-129-5p in M2 macrophages can be transmit to pulmonary fibroblasts via EVs and target FAM13A and STAT1, exerting a promotive effect on fibroblast proliferation during the PF development [54, 55]. In contrast, elevated sputum EVs that mainly come from macrophages exert a protective effect against pulmonary fibrosis progression. These antifibrotic miR-142-3p carried EVs can be transported to alveolar epithelial cells (AECs) and lung fibroblasts, inhibiting AECs epithelial-mesenchymal transition (EMT), and fibroblast activation [56]. Furthermore, lncRNA HOTAIRM1 delivered by AECs secreted EVs have been reported to activate lung fibroblasts and stimulate IPF through regulating the miR-30d-3p/HSF1/YY1 axis [57].

**Fig. 3:**
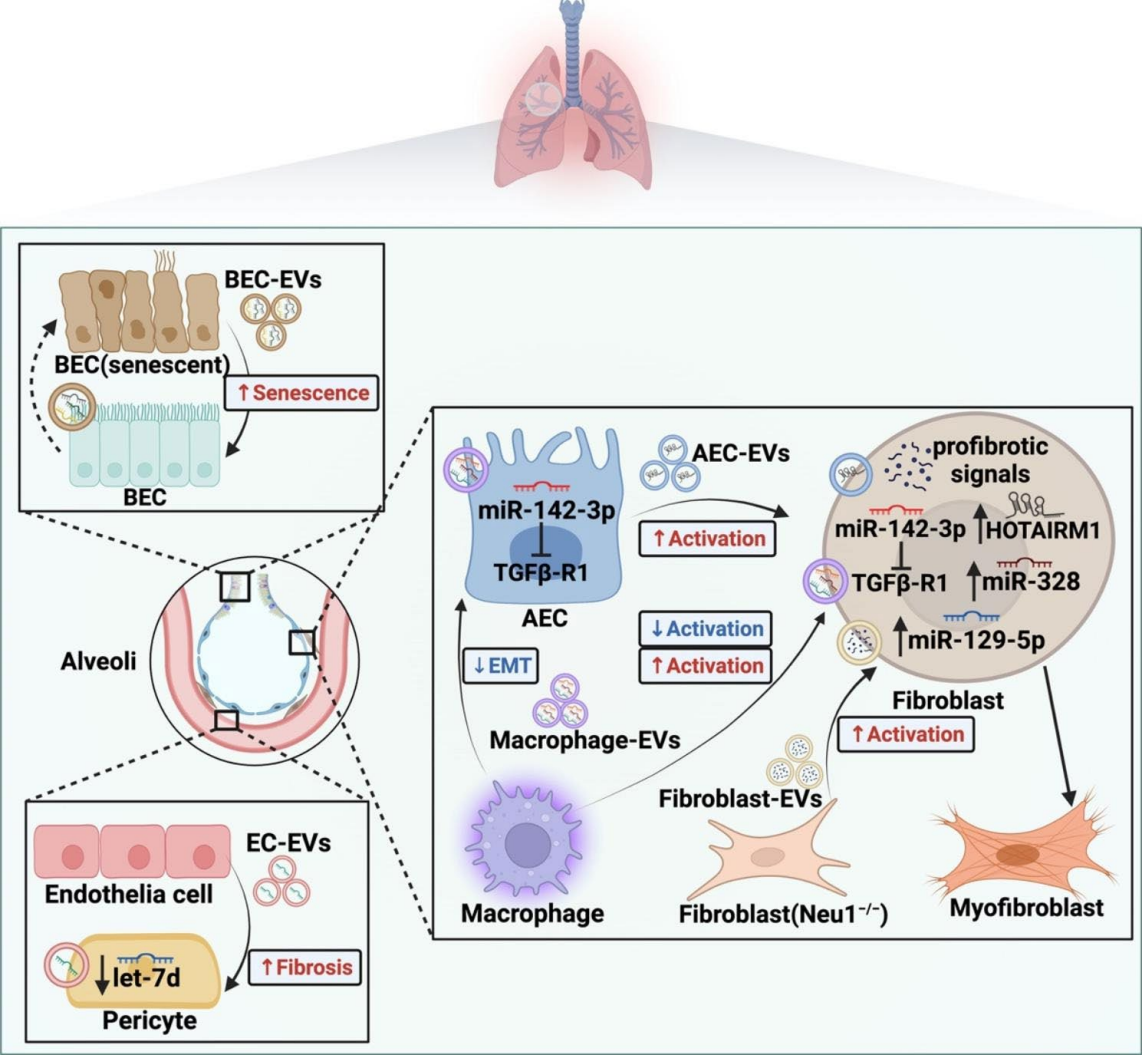
EV-mediated intercellular communication in fibrotic lung diseases. During lung fibrosis, a serious of cells in bronchi, alveoli, and capillaries promote diseases development through the delivery of pathological vesicles within each other. While macrophages are capable of both activation and suppression of fibroblasts via transferring different microRNA cargos

Fibroblasts are not only fibrogenic effector cells, but also can propagate fibrosis by secreting pathogenic EVs. Namely, neuraminidase 1(NEU1)-deficient fibroblasts cells, which share the phenotype of myofibroblast, can secrete numerous EVs loaded with activated components of the transforming growth factor (TGF)-β and WNT signaling pathways and induce fibrotic features in normal fibroblasts [58], indicating that the down regulation of NEU1 in IPF may be responsible for the overexpression of EVs and activation of profibrotic signals cascade. Despite pathological changes in a variety of cells, the activation of fibroblast plays a determinant role in pulmonary fibrosis (Fig. 3). It is worth noting that the function of cells changes dynamically during the disease progression, and that may account for the conflicting roles of macrophages mentioned above.

#### EVs as diagnostic and prognostic biomarkers in lung fibrosis

EVs in sputum and BALF (BALF-EVs) are attractive biomarkers for lung diseases due to their accessibility and non-invasiveness. A panel of miRNAs (miR-142-3p, miR-33a-5p, let-7d-5p) carried by sputum EVs of patients with IPF has been proposed as potential biomarkers due to high correlation with disease severity. The diagnostic efficiency AUC reaches 0.978 [59]. In BALF, EVs with special markers (CD56, CD105, CD142, CD31 and CD49e) can be detected to distinguish IPF from sarcoidosis and hypersensitivity pneumonitis [60]. In addition to fibrosis promotion, WNT-5a carried EVs overexpressed in BALF of IPF patient may serve as suitable candidates for PF diagnosis [50]. Tang et al. [61] analyzed the expression of non-coding RNAs (ncRNAs) in BALF-EVs from mice with mechanical ventilation-induced pulmonary fibrosis (MVPF) and identified thousands of differentially expressed ncRNAs compared with control group, suggesting that deep exploration of BALF-EVs for diagnosis has broad prospects. Interestingly, EVs in urine have shown promise in identifying lung fibrotic diseases, as they express miRNAs (miR-let-7d, miR-29a-5p, miR-181b-3p and miR-199a-3p) consistent with previous reports on miRNA expression in lung tissue/serum from IPF patients, which also indicate that IPF may have a systemic feature [62]. However, more efforts should be made to develop a simpler and faster detection method for EV and make it suitable for clinical application.

### Cardiovascular fibrosis

#### Pathological effects of EVs in cardiovascular fibrosis

Cardiac fibrosis is a common pathological feature of maladaptive remodeling and consist in many chronic heart diseases, including myocardial infarction (MI), dilated cardiomyopathy, and hypertension induced hypertrophic cardiomyopathy [63]. Under pathological conditions such as hypoxia, hypertension, metabolic abnormalities, and inflammation, cardiac fibroblasts (CFs) can be transformed into myofibroblasts and induce ECM deposition [64], which is the main driver of cardiac fibrosis. Cardiomyocytes have been reported to play a pivotal role in the phenoconversion of CFs by transferring miRNA loaded EVs (miR-208a and miR-92a)[65, 66]. During the acute phase of heart failure, increasing amounts of miR-30d-containing EVs are released by cardiomyocytes, which suppress proliferation and activation of CFs via directly targeting integrin α5 (ITGA5). However, the downregulation of miR-30d in EVs is responsible for cardiac fibrosis during the remodeling phase of HF [67]. Crosstalk between immune cells and CFs mediates fibrotic repair as well. In case of MI, EVs derived from activated CD4 positive (CD4+) T cells carrying miR-142-3p evoke cardiac fibroblasts activation and aggravate cardiac fibrosis by regulating APC/WNT signaling axis [68]. Similarly, EVs from macrophages show pro-fibrotic effect in the process of cardiac remodeling post-MI through the transmission of circUbe3 targeting miR-138-5p/RhoC axis [69]. Different from the one-to-one cellular communication reported in the above studies, epicardial fat (eFat)-derived EVs, which harbor a unique proinflammatory and profibrotic characteristic under the pathological state of atrial fibrillation, can target a broad range of atrial cells (such as cardiomyocytes, mesenchymal stem cells (MSCs), and endothelial cells) and deliver various profibrotic molecules, resulting in atrial fibrosis (AF) and atrial fibrillation [70].

**Fig. 4:**
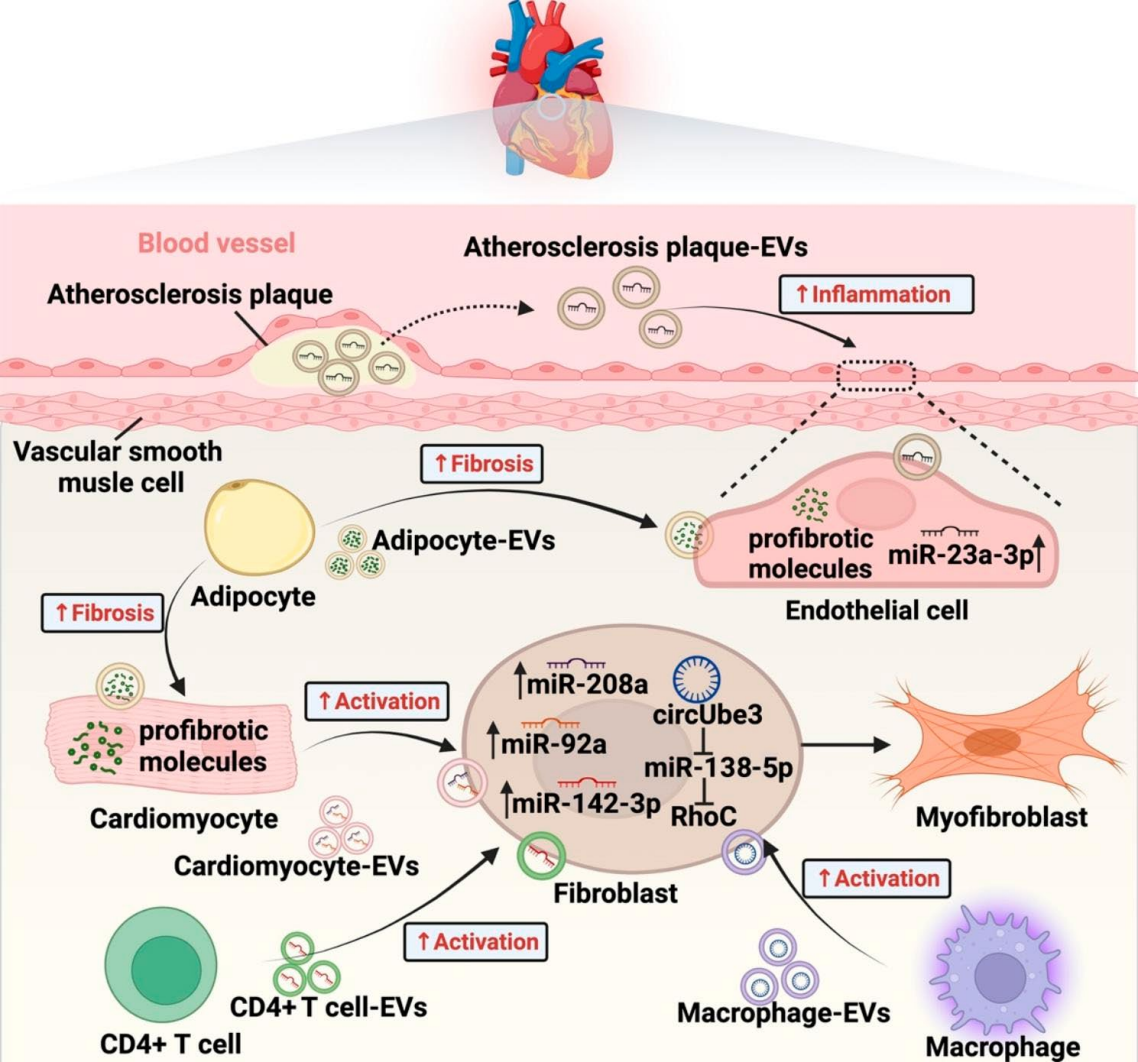
EV-mediated intercellular communication in cardiovascular fibrosis. Under pathological conditions, adipocytes secreted EVs exacerbate cardiac scarring via delivering profibrotic signals to endothelial cells and cardiomyocytes. Meanwhile fibroblasts can be activated by EVs from cardiomyocytes and immune cells through different cargos. In the blood vessels, pathological EVs released by atherosclerotic plaques spread atherogenesis to distant tissues

Atherosclerosis (AS) is the main cause of coronary heart disease and peripheral vascular disease. As a fibrotic and calcific lesion in the vessel wall, atherosclerosis plaques release EVs loaded with miR-23a-3p into the bloodstream and accelerate atherogenesis in remote locations by promoting endothelial inflammation [71]. Meanwhile, circulating endothelial cells derived EVs in obese/hypertensive could be involved in cardiovascular diseases by inducing cardiomyocyte hypertrophy and fibrosis [72] (Fig. 4). These findings emphasize the systemic pathogenicity of circulating EVs, which can transmit pro-fibrotic signals to more distant tissues after being secreted by damaged tissues into the blood.

#### EVs as diagnostic and prognostic biomarkers in cardiovascular fibrosis

There is mounting evidence that EVs can be used to diagnose myocardial fibrosis [73, 74]. Wang et al. [75] identified that the level of EV carried miR-425 and miR-744 in plasma could represent the progression of fibrosis in HF. Atherosclerotic conditions promote the packaging of miR-23a-3p [71] and miR-92a-3p [76] into EVs, which can be captured in plasma and reflect coronary artery disease. Upregulated lncRNA MIAT-rich EVs in serum samples of AF patients have been identified [77], providing a possible biomarker for AF diagnosis. Additionally, a decrease in HSP70 expression on serum EVs may connect with the progression of cardiac fibrosis [78]. Though abundant blood supply of the heart provides exceptional advantage for the diagnosis of cardiovascular fibrosis with circulating EVs, the fact that they are quickly cleared from circulation making it difficult to capture them timely.

### Renal fibrosis

#### Pathological effects of EVs in renal fibrosis

**Fig. 5:**
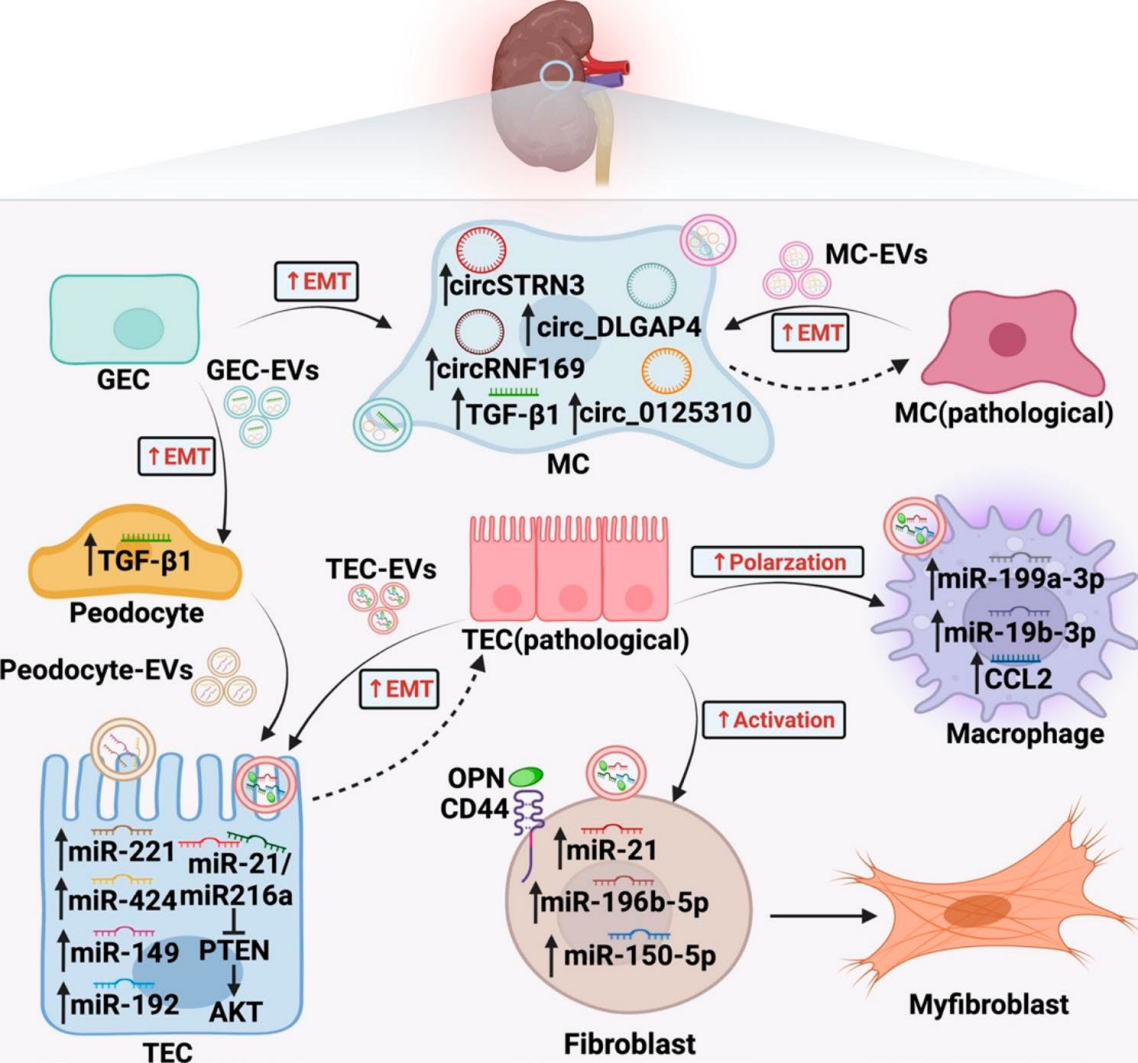
EV-mediated intercellular communication in renal fibrosis. In fibrotic kidney, EVs establish an intricate signal transmission network within renal cells and promote their pathological changes. TECs are the key point during fibrosis process due to the capability of immune regulation and fibroblast activation

Triggered by injured renal tubules, renal fibrosis occurs at the late stage of chronic kidney disease (CKD) and appears glomerulosclerosis, tubulointerstitial fibrosis (TIF), and angiosclerosis [79]. Accumulating evidence indicates that EVs can be released by almost all segments of kidney and mediate renal fibrosis by inducing damage of resident kidney cells, recruiting inflammatory cells, and activating fibroblasts [80, 81] (Fig. 5).

Renal autochthonous cells mainly encompass renal tubular epithelial cells (TECs), podocytes, glomerular endothelial cells (GECs), and mesangial cells (MCs), among which a complex network of signal transduction are constructed by EVs. Specifically, EVs from HG-stimulated GECs have been shown to introduce EMT and pro-fibrotic response in podocytes and MCs by delivering TGF-β1 mRNA [82, 83]. Ling et al. [84] demonstrated that circRNA (circRNF169 and circSTRN3) in EVs from GECs are also responsible for the damage effects on MCs. Research has also shown that increased EVs of MCs enriched for circ_DLGAP4 and circ_0125310 promote other normal cells proliferation and renal fibrosis via modulating miR-143/ERBB3/NF-κB/MMP-2 and miR-422a/IGF1R/p38 axis respectively in diabetic nephropathy [85, 86]. Furthermore, TECs treated with EVs from differentiated podocytes produce large amounts of ECM proteins accompanied by increased p38 and Smad3 phosphorylation [87]. Recent research has revealed that podocytes derived EVs mediate TECs damage partially through EV-contained miRNA cargos, such as miR-221 [88], miR-424 and miR-149 [89].

TECs are considered to play a central role in the renal fibrosis through exerting paracrine effects on many other kidney cells and then triggering a chain reaction [90]. Injured TECs not only overexpress fibrosis-related genes [91], but also transfer the phenotype of adjacent epithelial cells and trigger a vicious cycle of renal fibrosis. Notably, EVs derived from TGF-β-stimulated TECs carrying miR-21 and miR-216a have been found to induce neighbor cells undergo EMT by activating PTEN/AKT pathway, thereby promoting the progress of renal interstitial fibrosis [92, 93]. On the other hand, Jia Y et al. [94] reported that reducing the transfer of miR-192 by EVs from high glucose (HG)-induced TECs toward normal cells effectively protect renal cells and alleviate renal fibrosis in diabetic kidney disease (DKD). EVs derived from TECs also exert potent effect on driving persistent inflammation and fibroblast activation. In the kidney, macrophages are prominent inflammatory cells. It has been proved experimentally that EVs from damaged TECs contain more CCL2 mRNA, which recruitment macrophages and enhance inflammatory response [95]. Recent research has found that EVs released by albumin injured TECs induce kidney macrophage M1 polarization through the delivery of miR-199a-5p [96]and miR-19b-3p [97]. Furthermore, various studies point out that TECs transmit increased numbers of EVs containing miR-196b-5p, miR-21, miR-150-5p and osteopontin (OPN) to fibroblasts and promote its activation, thus aggravating interstitial renal fibrosis [98–101]. Together these data suggest that in renal fibrosis the intercellular communication centered on TECs, and inhibiting the EVs release from TECs seems to be an attractive therapeutic strategy.

#### EVs as diagnostic and prognostic biomarkers in renal fibrosis

EVs in urine are considered as effective non-invasive markers for the diagnosis of CKD. A series of dysregulated miRNAs and mitochondrial tRNAs (mtRNAs) in TECs-EVs, potential major source of urinary EVs, are correlated with the fibrotic status in an in vitro model of CKD [102]. Research by Petzuch et al. [103] showed that some increased miRNAs in urinary EVs are implicated in renal inflammation, fibrosis, and glomerular injury, which advance the application of urinary EVs as markers of renal fibrosis. Recently, hundreds of differentially expressed circRNAs have been revealed in CKD patients with or without renal fibrosis, in which the expression of circ_0036649 can reflect the score of TIF and glomerular sclerosis at a cut-off value of 0.597 with a sensitivity of 45.5% and specificity of 87.9%[104]. Circ_0008925, overexpressed in urinary EVs from glomerular disease patients, can diagnose renal fibrosis at a cut-off value of 0.093 with a sensitivity of 52.2% and specificity of 96.4%[105]. Despite the extensive coverage of EV-based diagnosis in renal disease [106], most of these reported biomarkers are connected with renal damage, but are incapable of directly reflecting the level of fibrosis. The understanding of how EVs regulate intra-nephron communication as well as their potential to reflect the degree of fibrosis in renal pathology need further study and future clinical trials.

From the above discussion, we can preliminarily believe that EVs play an important role in fibrotic diseases and have potential as diagnostic markers. The solution of clinical problems in the process of fibrosis has puzzled us for a long time. In recent years, more interest has focused on whether EVs can prevent and treat fibrosis. How to improve the anti-fibrosis of EVs has become a new research field.

## EV-based therapy in fibrosis disorders

The progressive deposition of ECM in fibrotic diseases leads to increased scarring of injured tissue, further promoting organ dysfunctions and even causing death [107, 108]. Numerous small molecules or compounds such as pirfenidone, nintedanib and PRI-724 have been applied in clinical trials for fibrosis. Most of them can only delay fibrotic process but cannot reverse or block the occurrence of fibrosis. What is worse, some drugs even have severe side effects [109, 110]. So, it is urgent to explore new targeted therapies for fibrosis. Encouraging results of EV administration from different origins, including stem cells, mature somatic cells, lung cells, and serum indicates that EVs play an important role for the treatment of fibrotic diseases (Table 2).

**Table 2:** Anti-fibrotic effect of EVs in different models

Origin	Cargos in EVs	Model	Mechanism	Function	Ref.
BMMSCs	undefined	mouse cGVHD	↓Th17 cells, ↑Treg	prolong the survival, diminish the clinical and pathological scores, ameliorate fibrosis in the skin, lung, and liver	[120]
BMMSCs	miR-34c-5p	mouse UUO	↓core fucosylation	reduce renal interstitial fibrosis	[124]
BMMSCs	MFG-E8	rat UUO	↓RhoA/ROCK pathway	reduce pathological changes and renal fibrosis	[126]
hucMSCs, BMMSCs	undefined	mouse BPD	modulate the pulmonary macrophage phenotype from “M1-like” to “M2-like”	restore lung architecture, decrease pulmonary fibrosis	[121, 122]
hucMSCs	CK1δ/β-TRCP	rat UUO	↑YAP degradation	lessen collagen deposition, alleviate renal fibrosis.	[125]
PL-MSCs	miR-29c	mouse DMD	↓myoblast differentiation, ↓TGF-β	decrease fibrosis in the diaphragm and cardiac muscles	[123]
CDCs	undefined	rat Aging	undefined	attenuate left ventricular hypertrophy and fibrosis, improve diastolic function	[128]
CDCs	miR-146a-5p	Pig DCM	undefined	promote cardiomyocyte proliferation, enhance angiogenesis, reduced myocardial fibrosis. reduce myocardial fibrosis	[129]
CDCs	miR-4488	mouse ACM	↓NF-κB	improve cardiac function, reduce cardiac inflammation and fibrosis, suppress arrhythmogenesis	[130]
CDCs	undefined	pig MI	undefined	decrease scarring, halt adverse remodelling and improve left ventricular ejection fraction	[131]
CDCs	undefined	pig VA	undefined	decrease myocardial scar, suppress slowly conducting electrical pathways, inhibit VA	[132]
CDCs	undefined	rat MI	undefined	transform fibroblast phenotype from inert cells to therapeutically active cells, reduce scar mass	[133]

**Table 2:** (continued)

Origin	Cargos in EVs	Model	Mechanism	Function	Ref.	
HLSCs	proteins	mouse NSAH	undefined	improve liver function and morphology, reduce liver fibrosis and inflammation	[136]	
LSCs	proteins and miRs	mouse/rat PF	undefined	reestablish normal alveolar structure, decrease collagen accumulation and myofibroblast proliferation	[139]	
hAECs	proteins and miRs	mouse PF	undefined	reduce lung inflammation, improve tissue-to-airspace ratio and reduce fibrosis	[142]	
iPSC-CMs	miR-106a–363 cluster (miR-106a, -18b, 19b, -20b, -92a, and − 363)	mouse MI	↓Notch3	induce cardiomyocyte cell proliferation, reduce myocardial fibrosis	[145]	
EnCs/ EpCs-II	miR-223/miR-27b-3p	mouse ALI/PF	↓RGS1	regulate immune balance of alveolar macrophages, reduce pulmonary fibrosis	[148]	
HBECs	miR-16, miR-26a, miR-26b, miR-141, miR-148a, and miR-200a	mouse PF	↓TGF-β-WNT crosstalk	inhibit myofibroblast differentiation and lung epithelial cellular senescence, reduce lung fibrosis	[149]	
HSCs	miR-214	mouse liver fibrosis	↓CCN2	inhibit fibrogenic signaling	[150]	
Macrophages	miR-233	mouse NSAH	↓TAZ	attenuate liver inflammation, reduce pro-fibrotic genes	[152]	
serum	miR-34c, -151-3p, -483-5p, -532-5p and - 687	mouse liver fibrosis	undefined	suppress hepatic collagen deposition and inflammation, reduce fibrosis	[153]	

### Stem cell derived EVs

With the characteristic of self-renewal and differentiation, stem cells are good candidates for regenerative medicine. Stem cell derived EVs, including MSCs, cardiosphere-derived cells (CDCs), human liver stem cells (HLSCs), lung spheroid cells (LSCs), human amniotic epithelial cells (hAECs) and human induced pluripotent stem cells (iPSCs), have been widely studied and show good therapeutic potential in many fibrotic diseases.

#### MSCs

MSCs are a subset of stromal cells and can be isolated from a variety of human tissues and organs, such as adipose tissue, bone marrow, placenta, and umbilical cord [111–115]. With the properties of immunomodulatory, anti-inflammatory and antioxidant, MSCs have been considered as promising therapies for multiple diseases [116], including fibrosis. Many studies have demonstrated that besides cell engraftment, MSCs can achieve their capacity of restoration through paracrine mechanisms including the secretion of EVs [117, 118]. EVs derived from MSCs (MSC-EVs) have been shown to inhibit inflammatory reaction, reduce fibrosis, and recover damaged tissue structure in many disease models. For example, our research found that human umbilical cord derived EVs (hucMSC-EVs) could effectively ameliorate liver fibrosis of CCl4-induced mice models [119]. EVs secreted by human bone marrow MSCs (BMMSC-EVs) attenuated fibrosis in the skin, lung, and liver by suppressing Th17 cells and inducing Treg in chronic graft-versus-host disease (cGVHD)[120]. Restored lung architecture and decreased pulmonary fibrosis have been observed in the model of bronchopulmonary dysplasia (BPD) after human Wharton’s Jelly-derived MSC-EVs or BMMSC-EVs treatment and its mechanism is partly associated with macrophage immunomodulation [121, 122].

Increasing evidence indicates that the therapeutic effects of EVs may attribute to their loaded miRNAs and proteins. MiR-29c in EVs secreted by placenta-derived MSCs (PL-MSCs) alleviates fibrosis in the diaphragm and cardiac muscles in duchenne muscular dystrophy (DMD)[123]. In addition, BMMSC-EVs enriched with miR-34c-5p can reduce fibrosis-related cell activation and renal interstitial fibrosis by inhibiting core fucosylation, a regulator of multiple profibrotic signaling pathways [124].The proteins transferred by EVs also play critical role in the treatment of fibrotic diseases. In unilateral ureteral obstruction (UUO) model, CK1δ/β-TRCP and MFG-E8 derived from MSC-EVs attenuate renal fibrosis by mediating YAP degradation and inhibiting the RhoA/ROCK pathway respectively [125, 126]. MSC- EVs exert broadly protective effects against a wide range of fibrotic diseases through various molecular mechanisms. While few studies have focused on whether EVs derived from different sources show the similar therapeutic effects for the same fibrotic diseases. Moreover, these studies mentioned above are still in the preclinical stage and the most appropriate administration route and dosage of MSC-EVs remain undefined.

#### Other stem cells

CDCs, a kind of cardiac progenitor cells, were found as an ideal therapeutic candidate for regenerative therapy over the past decade [127].Intra-cardiac injections of CDCs improve diastolic function and attenuate myocardial fibrosis in senescent and dilated cardiomyopathy model(DCM), during which process EVs secreted by cardiosphere-derived cells (CDC-EVs) mediate the beneficial effects via carrying miR-146a-5p [128, 129]. In arrhythmogenic cardiomyopathy (ACM) model, miR-4488 delivered by EVs from immortalized CDCs ameliorate underlying cardiac fibrosis by mitigating nuclear factor-κB (NF-κB) activation [130]. CDC-EVs also show strong anti-fibrotic effects and decrease myocardial scar after intramyocardial delivery in both MI and ventricular arrhythmias (VA)[131, 132]. EVs not only have a direct anti fibrosis effect on damaged tissues, but also can play a role in regulating the microenvironment. CDC-EVs can transform fibroblast phenotype and secretome from inert cells to therapeutically active cells, which reduce the scar mass of damaged heart after intramyocardially injection [133]. The biological safety of CDCs has been proved through clinical trials [129, 134], indicating that CDCs, including CDC-EVs, can be used as an effective treatment for cardiac scarring and even other fibrotic diseases.

Isolated from adult normal human liver, HLSCs share the characteristics of both mesenchymal stem cells and liver cells [135]. EVs released by HLSC significantly attenuate liver fibrosis and inflammation in a mouse model of NASH, which may attribute to the enriched EV proteins [136]. LSCs are therapeutic adult lung cells and express both lung epithelial and mesenchymal markers [137]. The disease-mitigating properties of LSCs have been revealed in a mouse model with bleomycin-induced pulmonary fibrosis [138]. Dinh et al. reported that inhalation of LSCs derived EVs (LSC-EVs) decrease both collagen deposition and myofibroblast proliferation in an IPF model, in which LSC-EVs exhibit superior therapeutic benefits than MSC-EVs [139]. HAECs, one of the perinatal stem cells isolated from the human placenta, show therapeutic potential for fibrotic diseases with differentiation capability and immunomodulatory properties [140, 141]. HAECs derived EVs (hAEC-EVs) have been reported to attenuate lung fibrosis in an IPF mouse model as well [142]. It is worth noting that combining anti-fibrotic drug serelaxin with hAEC-EVs enhance their therapeutic efficacy in pulmonary fibrosis [143].

IPSCs, a kind of reprogrammed somatic cells, share with embryonic stem cells the expression of marker genes and pluripotency [144]. EVs isolated from human iPSC-derived cardiomyocytes (iPSC-CMs) reduce myocardial fibrosis of ischemia-injured heart via miR‑106a-363 cluster-induced regulation of Notch3 signaling pathway [145].The application of EVs avoids the tumorigenesis risk of iPSCs and open a new door for therapeutic investigation [146]. However unstable genome is a great hindrance to the clinical application of iPSC and its EVs, which is also responsible for fewer studies related to iPSC-EVs therapy for fibrotic diseases than other stem cells.

### Mature somatic cells derived EVs

Respiratory system cells can release EVs, including lung epithelial cells, pulmonary vascular endothelial cells, fibroblasts, and many immune cells [147].The anti-fibrotic and regenerative properties of EVs derived from healthy adult lung cells have aroused wide interest in the treatment of pulmonary fibrosis. MiR-223 and miR-27b-3p transferred respectively by secreted EVs from vascular endothelial cells (EnCs) and type II alveolar epithelial cells (EpCs-II) regulate heterogeneity of alveolar macrophages by targeting regulator of G protein signaling-1 (RGS1), and thus reduce fibrosis in acute lung injury/acute respiratory distress syndrome (ALI/ARDS) and pulmonary fibrosis [148]. Moreover, human bronchial epithelial cell (HBEC) derived EVs loading miRNAs such as miR-16, miR-26a, miR-26b, miR-141, miR-148a, and miR-200a inhibit TGF-β-WNT pathway, leading to attenuation of pulmonary fibrosis in IPF [149].

In liver, HSCs derived EVs (HSC-EVs) have been proved to transfer miR-214 between hepatocytes and inhibit expression of CCN2, leading to the suppression of downstream fibrotic pathways [150]. Another finding, on the other hand, indicates that inhibition of HSC-EVs secretion has beneficial effects to prevent HSC activation and attenuate fibrosis in a mouse model of NASH [151]. In NAFLD/NASH, the activated myeloid-specific IL-6 signaling in macrophages promotes miR-233-enriched EVs secretion, which are then transported to hepatocytes and inhibit liver fibrosis by suppressing PDZ-binding motif (TAZ)[152]. Moreover, EVs isolated from normal mice serum also suppress hepatic fibrogenesis or fibrosis in liver injury models by its cargo miRNAs(microRNA-34c, -151-3p, -483-5p, -532-5p and - 687)[153]. In addition to canonical EVs, a kind of biotic nanodiscs with a diameter of 10-30 nm have been isolated and characterized in human blood and termed BNHBs, containing similar functional proteins with lipoproteins and EVs. BNHBs show strong biological prevention of pulmonary fibrosis by inhibiting the expression of α-smooth muscle actin and collagen-1 protein in a mouse model [154].Although EVs from somatic cells have shown beneficial effects in some fibrotic diseases, several issues must be carefully considered, including the standardization of donor cells and their anti-fibrotic effect on other organs or tissues apart from origins.

### Preconditioned and engineered EVs

#### Preconditioned EVs

Recent evidence shows that biological function of EVs is context dependent, and alter cell culture conditions or pretreatment are adaptive strategies to improve the efficacy of some therapeutic EVs, especially MSC-EVs [155]. Hypoxic precondition enhances paracrine effect of MSCs and the therapeutic potential of hypoxic induced MSC-EVs have been proved in a variety of diseases [156]. In a mouse model of MI, EVs secreted by hypoxia treated MSCs exhibit better effect on myocardial repair with smaller fibrotic scar and higher survival than normoxia-treated MSC-EVs via transferring more miR-210 [157]. A paper by Tolomeo et al. [158] showed that EVs from MSCs primed with pro-inflammatory cytokines induce anti-inflammatory phenotype of macrophages and reduce intestinal fibrosis in mice with experimental colitis, however the beneficial effect were not observed in mice treated by MSC-EVs from standard culture conditions. Similar results have been shown by MSCs pretreated with tumor necrosis factor alpha (TNF-α), EVs released by which contain higher miR-146a and are more effective in suppressing urethral fibrosis than that from untreated MSCs by inhibiting fibroblast activation and inflammatory responses [158]. As a result, these data indicate that inflammatory stimuli enhance the immune modulatory activity and repair efficacy of MSCs.

#### Engineered EVs

**Fig. 6:**
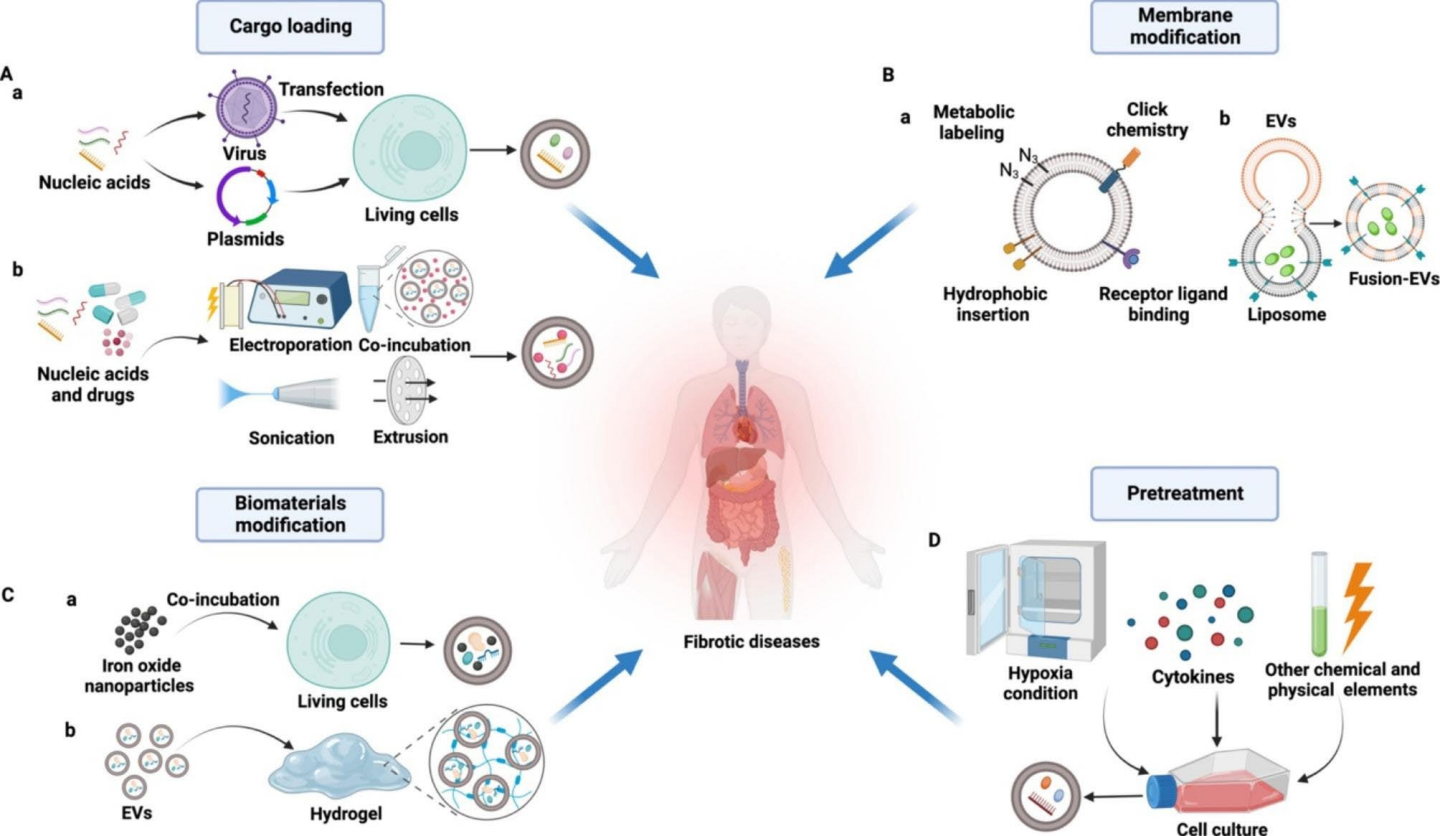
Precondition and engineering strategies of EV-based fibrosis therapy. (**A**) Viral vectors or plasmids are often used as endogenous loading approach to express genes of interest in living cells, which can promote expression of proteins, nucleotides, or other molecules. These components in cells can then be packed into EVs(a). Exogenous cargos such as can be directly transported into EVs by electroporation, incubation, ultrasound, or extrusion(b). (**B**) The membrane of EVs can be modified by metabolic labeling, click chemistry, hydrophobic insertion, and ligand binding(a). Liposome mediate membrane fusion endows EVs with specific contents and ligands(b). (**C**) Iron oxide nanoparticles incubated with living cells can be taken and encapsulated into EVs, which are able to be guided by magnetism(a). Combination with hydrogel enables EVs local delivery(b). (**D**) Pretreat cells by hypoxia, cytokines, and some other chemical and physical elements change the physiological characteristic of EVs secreted by them

Engineered EVs have been widely explored to overcome the shortcomings of natural EVs and improve their therapeutic efficiency. Coated with robust phospholipid membrane, EVs are good carriers for theranostic entities, such as proteins, nucleotides, lipids, drugs, and some functional nanoparticles [160]. Appropriate modification of EVs is an effective approach to improve their intrinsic biocompatibility, targeted delivery capability, biological activities and stability in circulation [161]. Cargo loading, membrane modification, and combination with biomaterials are the most common engineering strategies for EVs in the therapy of fibrotic diseases (Fig. 6).

Cargo loading strategies are widely used to increase theranostic contents inside or outside EVs, thus enhancing therapy capacity. Exogenous genes of interest usually transfected into donor cells by plasmids or viral vectors, where they express target nucleotides or proteins and encapsulated inside EVs. Many improved strategies have been developed to enhance target contents loading efficiency, such as methods termed exosomes for protein loading via optically reversible protein–protein interactions (EXPLORs)[162] and Targeted and Modular EV Loading (TAMEL) platform [163]. Chen et al. [164] transferred glial cell line-derived neurotrophic factor (GDNF) into adipose derived MSCs (ADSCs) and then isolated EVs enriched with GDNF, which reduce tubulointerstitial fibrosis better than natural ADSC derived EVs (ADSC-EVs) by activating SIRT1/eNOS signaling pathway in the UUO mouse model. Another research isolated EVs from ADSCs engineered to over express miR-181-5p and found that these miR-181-5p enriched EVs prevent liver fibrosis in vivo and vitro [165]. BMMSC-EVs packaged with CFZF-VPR, a zinc finger activator, successfully activated cystic fibrosis transmembrane conductance regulator (CFTR) expression in target cells, suggesting that these engineered EVs can be used as an adjunctive cellular therapeutic approach to treat cystic fibrosis [166]. Wang et al. [167] constructed miR-29 encapsulated EVs, on the surface which membrane protein Lamp2b is fused with the targeting peptide RVG. These EVs target kidney by RGV and then inhibit TGF-β3 by miR-29, reducing renal fibrosis effectively. Though gene transfection mediate cargo loading maintains the integrity of EVs, the low loading efficiency can not be ignored. By contrast, membrane penetration strategies such as electroporation, sonication, mechanical extrusion, and co-incubation are more direct methods for some nucleotides (microRNA and small interfering RNA) and drugs encapsulation.

EV surface functionalization is considered as an effective approach to enhance their target delivery and visual tracing capacity. There are multiple membrane modification methods for EVs like covalent modification (metabolic labeling and click chemistry), non-covalent modification (receptor ligand binding, hydrophobic interaction, and multivalent electrostatic interactions)[168]. In a mouse model of liver fibrosis, vitamin A derivative has been incorporated into the membrane of bare ADSC-EVs and effectively target activated HSCs, leading to inhibition of fibrotic cascade [169]. The liposome mediated EVs membrane fusion can not only change the contents of the vesicles but also endow them with specific receptors. Sun et al. [170] combined multiple engineered strategies and constructed efficient drug delivery system, where clodronate loaded liposome were fused with fibroblast-derived EVs (EL-CLD) and further loaded with Nintedanib to enhance anti-fibrotic effects. The EL-CLD hybrid system can accumulate in the fibrotic lung by depleting Kupffer cells and homing to fibrosis region, providing efficient orientation for fibroblast specific therapy.

Characterized by biocompatibility, chemical stability and good mechanical performance, biomaterials are being explored in modifying EVs to remedy the limitations of bare EVs and improve their therapeutic efficacy. For example, our previous study decorated neutrophil-derived EVs with superparamagnetic iron oxide nanoparticles (SPIONs) and achieved achieve higher tumor-targeting therapeutic effect with applying an external magnetic field in BALB/c nude mice [171]. In a MI model, MSC derived EV-mimic nanovesicles (NVs) have been prepared by incorporating with iron oxide nanoparticle (IONP-NVs) and these magnetic NVs show enhanced retention in infarcted heart by magnetic guidance, resulting in improved therapeutic potency in reducing cardiac apoptosis and fibrosis [172]. Considering that bared EVs can be cleared in a short time by circulatory system, hydrogels have been widely employed as slow release strategy by EVs based therapy [173]. EVs combined with a thermoresponsive hydrogel (fluid at 4 °C and gelatinous at body temperature) can be injected locally and retain in the injured sites, which reduce fibrosis and promote wound healing in fistula models [174, 175]. This method not only solves the difficulties of EVs local administration, but also extends EV residence time in damaged tissues (Table 3).

**Table 3:** Effect of preconditioned and engineered EVs in fibrotic diseases therapy

Precondition/Engineering strategy	Origin	Method	Model	Function	Ref.
hypoxic	BMMSCs	culture MSCs under hypoxia condition (0.5% O2)	mouse MI	improve cardiac function and reduce scar size	[157]
cytokine Preconditioning.	BMMSCs	pretreat MSCs with IL1β, IL6 and TNFα	mouse colitis	induce macrophage M2 polarization, decrease dintestinal fibrosis and angiogenesis	[158]
cytokine preconditioning.	hucMSCs	pretreat MSCs with TNFα	rat urethral stricture	inhibit fibroblast inflammation, suppress urethral fibrosis and stricture	[159]
cargo loading	ADSCs	transfect GDNF into ADSCs via a lentiviral transfection system	mouse UUO	reduce tubulointerstitial fibrosis	[164]
cargo loading	ADSCs	transfect miRNA-181-5p to ADSCs	mouse liver fibrosis	inhibit HSC activation, reduce liver fibrosis	[165]
cargo loading	BMMSCs	transfect CFZF-VPR into BMMSCs via plasmid	human bronchial epithelial cells	activate CFTR	[166]
cargo loading	mouse satellite cells	transfect Lamp2b-RVG vector and miR29 into mouse satellite cells	mouse UUO	target kidney, inhibit TGF-β signaling pathway, alleviate renal fibrosis	[167]
membrane modification	ADSCs	modify the surface of ADSC-EVs with vitamin A derivative by phospholipid insertion	mouse liver fibrosis	target active HSCs, reverse the fibrotic cascade	[169]
combination with biomaterials	BMMSCs	extrude IONP-incorporated MSCs serially and obtain IONP-incorporated NVs	rat MI	improve the retention of EVs in the infarcted heart, reduce cardiac apoptosis, inflammation, and fibrosis	[172]
combination with biomaterials	ADSC/ murine stem cells	combinate EVs with a thermoresponsive hydrogel and inject locally at 4 °C	pig esophageal fistula/rat colon fistula	retain EVs in the entire fistula tract, decrease inflammatory response and fibrosis, increase angiogenesis	[174, 175]
cargo loading, membrane modification	fibroblast	fuse the membrane of clodronate loaded liposome with fibroblast derived EVs and load it with Nintedanib	mouse PF	deplete Kupffer cells, target fibroblast and accumulate in fibrotic lung, reduce lung fibrosis	[170]

Though a mass of studies demonstrates the effectiveness of EV-based therapy, few of them clarify the pharmacokinetics EVs. After administration in vivo, the distribution of EVs have been detected in various organs, including liver, spleen, kidney, and lung [176]. While human MSC-EVs were found to accumulate in liver 24 h after intravenous injection, suggesting that they may be cleared mainly by macrophage [177]. Engineering modification enhance the ability of EVs to target some special tissue, but it does not avoid their high accumulation in clearance organs, which limit the clinal application of EVs. Fibrosis always occurs at the end stage of diseases, considering the rapid clearance of EVs from body, the mechanism how they remain a persistent protective remain unclear. Thus, improving the specific and durable retention of EVs in vivo in an opportunity for future endeavors.

## Perspectives and challenges

So far, we have made significant progress in understanding the biological characteristics and functions of EV in different physiological and pathological systems. As natural nanocarriers of bioactive molecules from parental cells, EVs are effective mediators of inter-tissue communication and natural therapeutic agents for many complex diseases. The new clinical experimental evidence of EV as potential candidate for fibrotic diseases biomarkers and natural therapeutic intervention is increasing, but still in the early stages of development. One of the main reasons hindering research of EVs is the basic technical challenges of standard isolation and characterization protocols [178]. The existing EV separation methods have many unsolvable problems, such as loss of their activity, pollution of non-vesicular substances and time-consuming separation process [179]. What is more, grouping EVs either by size or immunoaffinity may result in overlap among the different EV subsets and even some other particles [180]. Therefore, most of the published studies focus on the function of a mixed EV subpopulations instead of a particular group [181].In addition, the production quality assurance of EVs from various sources requires normative document and standard requirements.

As disease biomarkers, EVs have been widely studied due to their bioactive cargos can reflect the status of the donor cells and tissues. In fibrotic diseases, several dysregulated vesicular proteins and nucleic acids expressed levels are associated with fibrosis progression. Some of these pathological EVs can be transferred into body fluids, such as blood and urine, allowing them to be captured, identified, and detected in liquid biopsy [182]. Whereas few research take into consideration of clinical or environmental factors, such as fasting state, medications, ethnicity, age, gender, and general lifestyle, etc., which can also influence EV counts/contents in addition to diseases itself [183]. Although strict sample collection/storage and EV separation are not easy to apply to large-scale clinical trials, EVs derived from a variety of MSCs and progenitor cells have shown promising therapeutic potential in fibrotic diseases. Regarded as natural nano therapy agent, EVs show good biocompatibility and non-toxicity side effect compared to conventional pharmacological therapy. Moreover, extensive exploration of engineered EVs makes them a potential next-generation nanomedicine treatment platform [184]. However, they are far from clinical application due to several hurdles, for instance, significant heterogeneity in EV populations, deficiency of uniform quality control (QC) criteria, and necessity of comply with national and international accreditation for new drugs development and administration [185]. In conclusion, advancement of basic research in EVs will promote the broader application of them in the diagnosis and treatment of fibrosis diseases in the future.

## Data Availability

Not applicable.

## Abbreviations

EVs: Extracellular vesicles
PM: Plasma membrane
MVs: Microvesicles
MVB: Multivesicular bodies
ISEV: International Society for Extracellular Vesicles
ECM: Extracellular matrix
HBV/HCV: Hepatitis B virus/ Hepatitis C virus
NAFLD: Non-alcoholic fatty liver disease
NASH: Non-alcoholic steatohepatitis
Akr1b7: Aldo-keto-reductase 1b7
HSCs: Hepatic stellate cells
TRAIL: Tumor necrosis factor-related apoptosis-inducing ligand
TLR3: Toll-like receptor 3
IL-17 A: Interleukin-17 A
CCl4: Carbon tetrachloride
MASP1: Mannan-binding lectin serine protease 1
LncRNA-H19: Long non-coding RNA-H19
SHP: Small heterodimer partner
AV+: Annexin V-positive
BECs: Bronchial epithelial cells
BALF: Bronchoalveolar lavage fluid
PF: Pulmonary fibrosis
MFPF: Mechanical ventilation-induced pulmonary fibrosis
IPF: Idiopathic pulmonary fibrosis
NEU1: Neuraminidase 1
ncRNAa: Non-coading RNAs
TGF: Transforming growth factor
AECs: Alveolar epithelial cells
EMT: Epithelial-mesenchymal transition
MI: Myocardial infarction
HF: Heart failure
CF: Cardiac fibroblast
ITGA5: Integrin α5
eFat: Epicardial fat
MSCs: Mesenchymal stem cells
AS: Atherosclerosis
AF: Atrial fibrosis
CKD: Chronic kidney disease
TIF: Tubulointerstitial fibrosis
TECs: Tubular epithelial cells
GECs: Glomerular endothelial cells
OPN: Osteopontin
DKD: Diabetic kidney disease
HG: High glucose
MCs: Mesangial cells
mtRNAs: Mitochondrial tRNAs
CDCs: Cardiosphere-derived cells
HLSCs: Human liver stem cells
LSCs: Lung spheroid cells
hAECs: Human amniotic epithelial cells
iPSCs: induced pluripotent stem cells
MSC-EVs: MSCs derived EVs
hucMSC-EVs: Human umbilical cord derived EVs
BMMSC-EVs: Bone marrow MSCs derived EVs
cGVHD: Graft-versus-host disease
BPD: Bronchopulmonary dysplasia
PL-MSCs: Placenta-derived MSCs
DMD: Duchenne muscular dystrophy
UUO: Unilateral ureteral obstruction
DCM: Dilated cardiomyopathy model
CDC-EVs: CDC-derived EVs
ACM: Arrhythmogenic cardiomyopathy
NF-κB: Nuclear factor-κB
VA: Ventricular arrhythmias
hAEC-EVs: hAECs derived EVs
iPSC-CMs: iPSC-derived cardiomyocytes
EnCs: Vascular endothelial cells
EpCs-II: Type II alveolar epithelial cells
RGS1: Regulator of G protein signaling-1
ALI/ARDS: Acute lung injury/acute respiratory distress syndrome
PF: Pulmonary fibrosis
HBEC: Human bronchial epithelial cell
HSC-EVs: HSCs derived EVs
GDNF: Glial cell line-derived neurotrophic factor
ADSCs: Adipose derived MSCs
ADSC-EVs: ADSC derived EVs
CFTR: Cystic fibrosis transmembrane conductance regulator
SPIONs: Superparamagnetic iron oxide nanoparticles
NVs: Nanovesicles
